# CD137 ligand reverse signaling skews hematopoiesis towards myelopoiesis during aging

**DOI:** 10.18632/aging.100588

**Published:** 2013-08-08

**Authors:** Qianqiao Tang, Liang Kai Koh, Dongsheng Jiang, Herbert Schwarz

**Affiliations:** ^1^ Department of Physiology, Yong Loon Ling School of Medicine, National University of Singapore, Singapore; ^2^ National University of Singapore Graduate School of Integrative Science and Engineering, Singapore; ^3^ Department of Dermatology and Allergic Diseases, University of Ulm, Germany

**Keywords:** Immunosenescence, CD137, aging-induced myelopoiesis, T cell, inflammation

## Abstract

CD137 is a costimulatory molecule expressed on activated T cells. Its ligand, CD137L, is expressed on the surface of hematopoietic progenitor cells, and upon binding to CD137 induces reverse signaling into hematopoietic progenitor cells promoting their activation, proliferation and myeloid differentiation. Since aging is associated with an increasing number of myeloid cells we investigated the role of CD137 and CD137L on myelopoiesis during aging. Comparing 3 and 12 months old WT, CD137^−/−^ and CD137L^−/−^ mice we found significantly more granulocytes and monocytes in the bone marrow of older WT mice, while this age-dependent increase was absent in CD137^−/−^ and CD137L^−/−^ mice. Instead, the bone marrow of 12 months old CD137^−/−^ and CD137L^−/−^ mice was characterized by an accumulation of hematopoietic progenitor cells, suggesting that the differentiation of hematopoietic progenitor cells became arrested in the absence of CD137L signaling. CD137L signaling is initiated by activated CD137-expressing, CD4^+^ T cells. These data identify a novel molecular mechanisms underlying immune aging by demonstrating that CD137-expressing CD4^+^ T cells in the bone marrow engage CD137L on hematopoietic progenitor cells, and that this CD137L signaling biases hematopoiesis towards myelopoiesis during aging.

## INTRODUCTION

Aging is a process accompanied by low grade chronic inflammation. In both, man and mouse, hematopoieisis shift from lymphopoiesis to myelopoiesis during aging. Not only do the numbers of myeloid progenitor cells exceed those of lymphoid progenitor cells but hematoptoietic stem cells also tend to develop a bias towards myeloid lineage differentiation [1-3]. This change in balance of myelopoiesis and lymphopoiesis has important clinical consequences because the impaired adaptive immunity renders elderly people susceptible to an increased tumor incidence as well as a reduced vaccine efficacy [4, 5]. With aging populations in many countries, and the appearance of new infectious diseases it is becoming increasingly important to understand the mechanisms behind immune aging so we may be able to reduce morbidity, mortality and medical expenses.

Various mechanisms have been proposed explaining the imbalance of lymphopoiesis and myelopoiesis in aged humans and animals. DNA damage and a deficiency in telomerase in hematopoietic stem cells have been related to the waning potential of lymphoid differentiation [6, 7]. A changed bone marrow microenvironment including a reduced support from stoma cells and increased levels of inflammatory cytokines were also linked to the dysregulated hematopoiesis [8, 9].

Recently, it was reported that polyfunctional memory CD4^+^ and CD8^+^ T cells accumulate in the bone marrow of aged persons and that the increase of these memory T cells in the bone marrow is mainly antibacterial and anti-tumor in nature [10]. Since T cells are able to secrete cytokines such as GM-CSF and G-CSF that support myeloid differentiation, it is possible that the accumulated T cells in the bone marrow bias the differentiation of hematopoietic stem cells towards the myeloid lineage [11]. Studies have shown that CD4^+^ T cells in particular are essential for maintaining myelopoiesis. Deletion of CD4^+^ T cells, but not of CD8^+^ T cells, impairs myelopoiesis in the bone marrow [12, 13]. Aside from soluble factors produced by T cells, surface molecules expressed on the T cells may also influence hematopoiesis by interacting with their ligands on the hematopoietic progenitor cells.

CD137 is one of the costimulatory molecules expressed on T cells upon activation [14] and released by activated T cells as a soluble molecule [15, 16]. Its ligand, CD137L, is expressed on the surface of antigen presenting cells (APC) and hematopoietic progenitor cells, and CD137L reverse signaling enhances proliferation and differentiation of human and murine hematopoietic progenitor cells as well as of monocytes [17-21]. More recently, we reported that the frequency of CD137^+^ CD4^+^ T cells is increased in the bone marrow of mice experiencing acute inflammation or infections, and that these T cells induce myeloid differentiation of the bone marrow cells [22]. Therefore, we hypothesized that the accumulation of T cells in the bone marrow of aged animals may contribute to the skewing of hematopoiesis towards myelopoiesis, and that differentiation of hematopoietic progenitor cells is influenced by reverse signaling of CD137L. Examining the levels of myelopoiesis in aged WT, CD137^−/−^ and CD137L^−/−^ mice, we found fewer mature myeloid cells but more hematopoietic progenitor cells in the bone marrow of the two knockout strains. In a coculture of activated WT CD4^+^ T with WT or lineage^−^ hematopoietic progenitor cells less myeloid differentiation was observed when the progenitor cells lacked CD137L. These findings suggest a role of CD137L reverse signaling in driving myelopoiesis during aging.

## RESULTS

### CD137L reverse signaling enhances myelopoiesis during aging

**Figure 1:**
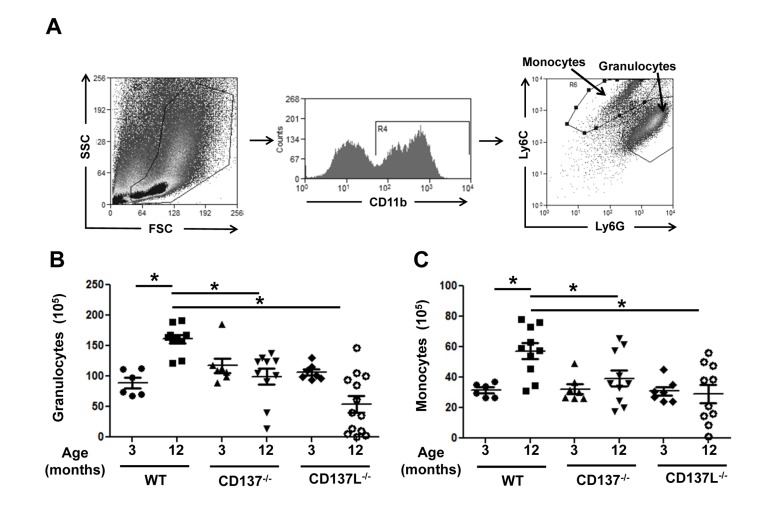
Aged CD137^−/−^ and CD137L^−/−^ mice have reduced myelopoiesis in bone marrow compared to WT Bone marrow cells of 3 and 12 months old WT, CD137^−/−^ and CD137L^−/−^ mice were stained for CD11b, Ly6G and Ly6C. (**A**) Gating strategy for (**B**) granulocytes (CD11b^+^, Ly6C^+^, Ly6G^+^) and (**C**) monocytes (CD11b^+^, Ly6C^+^, Ly6G^−^) in the bone marrow. (**B**) Number of granulocytes and monocytes in the bone marrow. Each group consisted of at least 5 mice. Data are representative of two independent experiments. *p<0.05.

To assess the contribution of the CD137 receptor/ligand system on myelopoiesis during aging, we investigated the number of granulocytes and monocytes in the bone marrow in young and old mice. Bone marrow cells of 3 and 12 months old WT, CD137^−/−^ and CD137L^−/−^ mice were isolated and stained for CD11b, Ly6G and Ly6C. Granulocytes were categorized as CD11b^+^, Ly6C^+^, Ly6G^+^, and monocytes were categorized as CD11b^+^, Ly6C^+^, Ly6G^−^ (Fig. 1A).

At the age of 3 months, WT, CD137^−/−^ and CD137L^−/−^ mice had similar numbers of granulocytes and monocytes. At the age of 12 months, the numbers of granulocytes and monocytes had increased 1.5-fold in WT mice. However, there was no corresponding increase in the numbers of granulocytes and monocytes in 12 months mice old CD137^−/−^ or CD137L^−/−^ mice, suggesting that aging-driven myelopoiesis depends on CD137 – CD137L interactions (Fig. 1B, C).

### During aging CD4^+^ T cells accumulate in the bone marrow

CD137 – CD137L interactions have been shown to mediate the increase of myelopoiesis during infections by CD137^+^ CD4^+^ T cells accumulating in bone marrow and activating hematopoietic progenitor cells and their myeloid differentiation [22]. We wondered whether there is a similar increase of CD137^+^ CD4^+^ T cells in the bone marrow of aged CD137^−/−^ and CD137L^−/−^ mice, and whether the missing increase in myelopoiesis in these mice is due to a deficiency in the CD137^+^ CD4^+^ T cells or in the hematopoietic progenitor cells.

**Figure 2:**
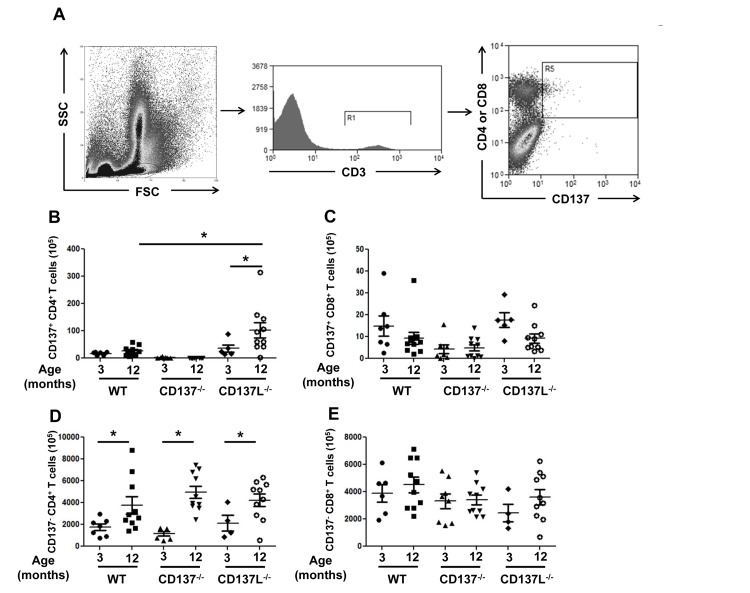
Increased numbers of CD4^+^ T cells in aged mice Bone marrow cells from 3 and 12 months old WT, CD137^−/−^ and CD137L^−/−^ mice were stained for CD3, CD137, CD4 and CD8. (**A**) Gating strategy of CD137^+^ T cells. (**B,C**) Numbers of (**B**) CD137^+^, CD4^+^ and (**C**) CD137^+^, CD8^+^ T cells. (**D,E**) Numbers of (**D**) CD137^−^, CD4^+^ and of CD137^−^, CD8^+^ T cells. Each group consisted of at least 5 mice. Data are representative of two independent experiments. *p<0.05.

Bone marrow cells from 3 and 12 months old WT, CD137^−/−^ and CD137L^−/−^ mice were stained for CD3, CD4, CD8 and CD137. CD137^−/−^ mice served as a negative control for the background staining of CD137 (Fig. 2A). There were very few CD137^+^ T cells in the bone marrow in young naïve mice which is in line with earlier observations [22]. As the WT mice aged, there was no significant increase in the number of CD137^+^ T cells for both CD4^+^ and CD8^+^ T cells. However, the number of CD137^+^ CD4^+^ T cells was significantly increased in 12 months old CD137L^−/−^ mice compared to the 3 months old CD137L^−/−^ mice as well as the 12 months old WT mice (Fig. 2B, C).

There was also a general increase in the number of CD137^−^ CD4^+^ T cells in the bone marrow of 12 months old mice in all three strains compared to their 3 months old controls. There was no significant difference in the numbers of CD137^−^ CD4^+^ T cells among the three strains of mice (Fig. 2D). The numbers of CD137^−^ CD8^+^ T cells remained unchanged (Fig. 2E).

### CD137L reverse signaling promotes differentiation of progenitor cells to mature myeloid cells during aging

Since the total numbers of CD4^+^ T cells in the bone marrow were similar between WT, CD137^−/−^ and CD137L^−/−^ mice, we wondered whether the reduced production of myeloid cells in the knockout strains was due to an unresponsiveness of the hematopoietic progenitor cells. Their numbers were therefore quantified in order to deduce whether a block of differentiation may be caused by the absence of CD137 or CD137L.

**Figure 3:**
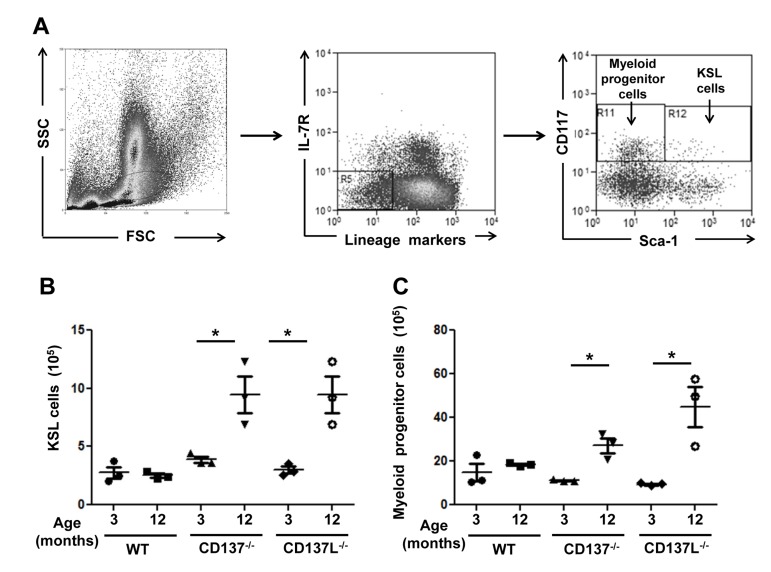
Increased numbers of myeloid progenitor cells in aged CD137^−/−^ and CD137L^−/−^ mice Bone marrow cells from 3 and 12 months old WT, CD137^−/−^ and CD137L^−/−^ mice were stained for lineage marker, IL-7R, CD117 and Sca-1. Cells were first gated on the progenitor cell region based on forward and side scatter. Then lineage marker^−^, IL-7R^−^ cells were gated out for analysis of myeloid progenitors (CD117^+^ Sca-1^−^) and KSL (CD117^+^ Sca-1^+^) cells. (**A**) Gating strategy of KSL cells and myeloid progenitor cells. (**B,C**) Number of (**B**) KSL cells and (**C**) myeloid progenitor cells in 3 and 12 months old mice. Each group consisted of at least 3 mice. Data are representative of two independent experiments. *p<0.05.

Bone marrow cells isolated from the 3 and 12 months old WT, CD137^−/−^ and CD137L^−/−^ mice were stained for the lineage markers Sca-1, CD117 (c-kit) and IL-7R. Hematopoietic stem and progenitor cells are referred to as KSL cells (CD117^+^, Sca-1^+^, IL^−^ 7R^−^, Lin^−^). Categorization of KSL cells and myeloid progenitor cells were based on the expression of Sca-1 and CD117 (Fig. 3A).

Numbers of KSL (Fig. 3B) cells and myeloid progenitor cells (Fig. 3C) did not differ significantly in 3 months old mice between the three strains. However, at the age of 12 months, both CD137^−/−^ and CD137L^−/−^ mice had increased numbers of KSL cells and myeloid progenitor cells in the bone marrow while the numbers of KSL cells and myeloid progenitor cells had remained constant in the WT mice (Fig. 3B, C). The data suggested that the lack of CD137L reverse signaling prevented hematopoietic progenitor cells from differentiating to mature granulocytes and monocytes.

**Figure 4:**
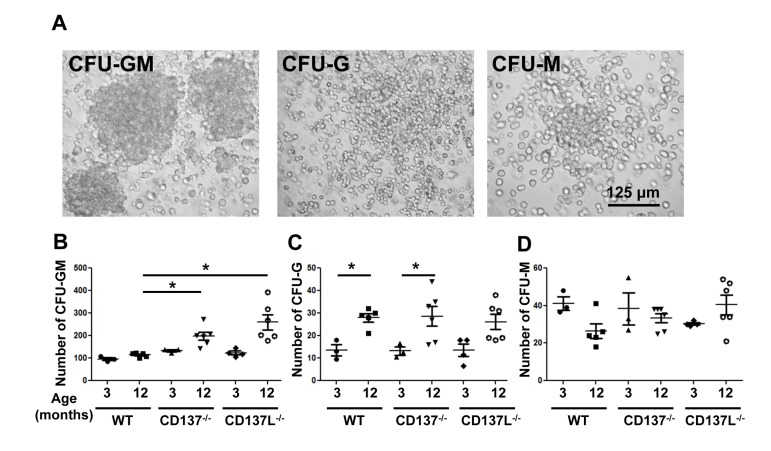
Numbers of colony forming units of the myeloid lineage (**A**) Morphology of CFU-Granulocyte-Macrophage (CFU-GM), CFU-Granulocyte (CFU-G) and CFU-Macrophage (CFU-M). Magnification: 20x. (**B-D**) The frequencies of colony forming units (CFU) are stated per 10^5^ total bone marrow cells: (**B**) CFU-GM, (**C**) CFU-G and (**D**) CFU-M. Each group consisted of at least 3 mice. Data are representative of two independent experiments. *p<0.05.

To confirm that the absence of CD137 or CD137L causes a differentiation block, we assessed the numbers of myeloid colony forming units (CFU) in the bone marrow of 3 and 12 months old mice. CFU-Granulocyte (CFU-G), CFU-Macrophage (CFU-M), CFU-Granulocyte/Macrophage (CFU-GM) were identified based on their morphologies (Fig. 4A).

The most abundant type of CFU was the CFU-GM which contains progenitor cells for granulocytes and monocytes. At the age of 3 months, WT mice had significantly fewer CFU-GM than CD137^−/−^ or CD137L^−/−^ mice (Fig. 4B), which is consistent with earlier findings showing more myeloid CFU in the young CD137^−/−^ mice compared to young WTmice [23]. At the age of 12 months the number of CFU-GM in the CD137^−/−^ mice had further increased from 133±9 to 198±44 per 10^5^ cells. The increase in 12 months old CD137L^−/−^ mice was even more substantial, from 123±17 to 256±84 per 10^5^ cells, which is almost a 2-fold increase. WT mice also had more myeloid progenitor cells at the age of 12 compared to 3 months, however, the difference was much smaller than in either of the two knockout strains, and had increased only from 96±11 to 114±10 per 10^5^ cells (Fig. 4B).

CFU-G and CFU-M represent more committed progenitor cells with a more restricted potential to differentiate. There were more CFU-G in 12 months old mice than 3 months old mice but there was no difference between WT and the two knockout strains (Fig. 4C). No significant change was detected for CFU-M in all three strains regardless of the age (Fig. 4D).

### CD137^+^ CD4^+^ T cells enhance myeloid cell differentiation of lineage^−^ hematopoietic progenitor cells

It has been previously shown that CD137^+^, CD4^+^ T cells can induce differentiation of lineage^−^ hematopoietic progenitor cells from young mice [22]. Therefore, we tested whether CD137L reverse signaling is also essential for the aged progenitor cells to differentiate to mature myeloid cells.

**Figure 5:**
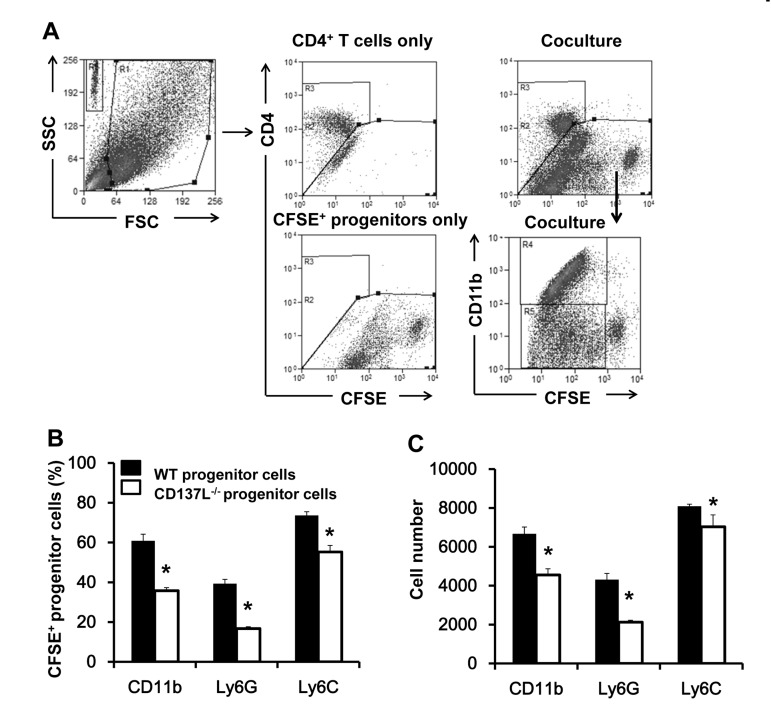
Increased proliferation and myeloid differentiation of WT progenitor cells Lineage^−^ hematopoietic progenitor cells were isolated from 12 months old WT and CD137L^−/−^ mice, labeled with CFSE, and cocultured with activated WT CD4^+^ T cells at a ratio of 1:2 for 6 days. Cells were then harvested and stained for CD4, CD11b, Ly6G and Ly6C. (**A**) Gating strategy of identifying CD4^+^ T cells and CFSE-labeled progenitor cells. (**B**) Percentages and (**C**) absolute numbers of CD11b^+^, Ly6G^+^ and Ly6C^+^ cells in the CFSE^+^ population. Quadruplicates were performed for each treatment group. Data are representative of two independent experiments. *p<0.05.

Lineage^−^ progenitor cells were isolated from bone marrow cells of 12 months old WT and CD137L^−/−^ mice. The cells were labeled with CFSE before being cocultured with activated WT CD4^+^ T cells for 6 days. To better distinguish the cell populations T cells were further stained for CD4. Percentages and absolute cell numbers for CD11b, Ly6G and Ly6C were analyzed in the CFSE^+^, CD4^−^ population (Fig. 5A).

WT and CD137L^−/−^ progenitor cells (CFSE^+^) responded differently to activated T cells in their differentiation. WT progenitor cells had a stronger preference than CD137L^−/−^ progenitor cells in differentiating to the myeloid lineage, as evidenced by the higher percentages of CD11b^+^(62.3±4.5 vs 33.9±1.8), Ly6G^+^ (38.2±2.2 vs 15.5±0.9) and Ly6C^+^ (47.2±2.2 vs 22.4±1.6) cells among the proliferating cells (CFSE^low^) (Fig. 5B, and Supplementary Figure 1). Accordingly, WT progenitor cells yielded also significantly higher absolute numbers of CD11b^+^(6667±347 vs 4552±319), Ly6G^+^ (4309±323 vs 2215±96), and Ly6C^+^ (8088±109 vs 7030±612) cells than the CD137L^−/−^ progenitor cells (Fig. 5C).

## DISCUSSION

The aging-related skewing of hematopoiesis towards myelopoiesis is a well-known phenomenon and has been linked to the development of various diseases including the increasing occurrence of infections and malignancies in older individuals. The role of proinflammatory molecules in driving myelopoiesis has been extensively investigated during the last decade [24, 25].

**Figure 6:**
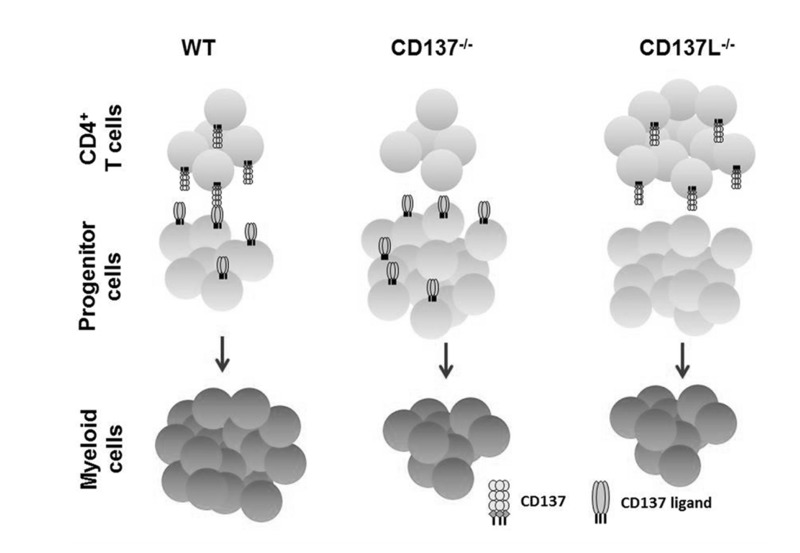
Schematic representation of CD137 – CD137L interactions in age-related myelopoiesis Depicted is the situation for 12 months old WT, CD137^−/−^ and CD137L^−/−^ mice. The absence of CD137 on activated CD4^+^ T cells or of CD137L on hematopoietic progenitor cells leads to a reduced number of myeloid cells in CD137^−/−^ and CD137L^−/−^ mice, respectively.

Previously, we have reported that CD137L signaling enhances myelopoiesis during inflammation caused by infections [22]. Since aging is also linked to an increased inflammatory state [4, 5] we wondered whether CD137L drives myelopoiesis during aging. The data show that CD137^−/−^ and CD137L^−/−^ mice at the age of 12 months do not experience a similar increase in the number of granulocytes and monocytes as WT mice, suggesting that the bias towards myelopoiesis depends on CD137 – CD137L interactions (Fig. 6).

Upon infection the numbers of CD137^+^ CD4^+^ T cells rapidly increased in the bone marrow [22] but we did not observe a similar increase in the number of CD137^+^ CD4^+^ T cells in 12 months old compared to 3 months old WT mice. This is probably due to the different degrees of inflammation the mice experienced. During acute peritonitis mice develop a strong inflammation within 48 h [22] while during aging mice have a much lower degree of chronic inflammation. The low numbers of CD137^+^ CD4^+^ cells are probably sufficient to maintain myelopoiesis during aging, while a fast expansion of CD137^+^ CD4^+^ T cells may only be required during emergencies, such as infections, when a more substantial increase in myelopoiesis is needed for pathogen clearance.

There was however, a significant increase in the number of CD137^+^ CD4^+^ T cells in 12 months old CD137L^−/−^ mice despite the lower level of myelopoiesis in the bone marrow of these mice. One potential explanation could be the reciprocal relationship between expression levels of CD137 and CD137L, where a decrease of CD137 expression leads to an increase in CD137 expression, and vice versa [26, 27]. An alternative explanation may be due to an unclosed feedback loop. Since CD137^+^ CD4^+^ T cells function as stimulators of myelopoiesis, and since a positive feedback signal in the form of more myeloid cells does not occur in the absence of CD137L signaling, the CD137^+^ CD4^+^ T cells continue to home to the bone marrow. The link between activated CD4^+^ T cells and myeloid differentiation of hematopoietic progenitor cells was confirmed by coculturing activated CD4^+^ T cells with WT or CD137L^−/−^ progenitor cells where the lack of CD137L signaling resulted in a reduced myeloid differentiation.

This unresponsiveness of CD137L^−/−^ progenitor cells also explains the lower myelopoiesis in CD137L^−/−^ mice despite the increased numbers of CD137^+^ CD4^+^ T cells. In contrast, the numbers of the more primitive KSL cells, myeloid progenitor cells and myeloid colony forming units were increased in 12 months old CD137^−/−^ and CD137L^−/−^ mice. These data suggest that the absence of CD137L reverse signaling causes a differentiation block in the progenitor cells, and hence arrests them at a less differentiated stage, leading to a lower number of mature granulocytes and monocytes in the bone marrow.

The potency of CD137L as a myeloid growth and differentiation factor may – at least in part – be due to the fact that CD137L can associate with other signaling molecules, such as TLR4, and potentiates TLR4 signaling [28]. CD137L also associates with TNFRI, utilizing the TNFRI signaling pathway [29]. These properties of CD137L allow for the possibility that it provides the nucleus for the formation of a larger signaling complex. The demonstrated translocation of NF-κB and activation of protein tyrosine kinases, ERK 1/2, p38 MAPK, MEK and PI3-K by CD137L signaling would be in line with this assumption [30].

Interestingly, naïve young CD137^−/−^ and CD137L^−/−^ mice have more myeloid cells, arguing for an inhibitory influence of CD137 – CD137L interactions on myelopoiesis [23, 31]. These data seem to contradict this study and earlier ones [17, 18, 20, 22]. An explanation may be that at a steady state CD137L reverse signaling serves as a negative regulator of myelopoiesis to prevent overproduction of myeloid cells while during inflammatory conditions it clearly enhances the number of myeloid cells. A similar seemingly conflicting role of CD137L was found in the activation of B cells where CD137L signaling leads to activation and proliferation *in vitro* [32, 33] while *in vivo* CD137L prevents hyperproliferation of germinal centre B cells, evidenced by the fact that aged CD137L^−/−^ mice tend to develop germinal B cell lymphoma [34].

This study expands our understanding of the hematopoietic activities of the CD137/CD137L system by demonstrating that reverse CD137L signaling initiated by CD137^+^, activated CD4^+^ T cells in hematopoietic progenitor cells not only drives myelopoiesis during infections but also in aging. It further identifies CD137L reverse signaling in hematopoietic progenitor cells as an underlying molecular mechanism of aging-associated myelopoiesis, and suggests CD137 and CD137L as potential targets for therapeutically directing hematopoiesis during disease and aging.

## METHODS

### Preparation of bone marrow cells, splenocytes and T cells

Mice were euthanized by CO_2_ inhalation. The femur bones were dissected and the bone marrow was flushed out aseptically with phosphate-buffered saline (PBS), 2 mM EDTA using a 10 ml syringe and 27G needle. Total bone marrow cells were passed through a 30 μm filter (Miltenyi Biotec, Bergisch Gladbach, Germany), washed with PBS containing 2 mM EDTA and resuspended in RPMI1640 medium (Sigma-Aldrich, St Louis, MO, USA), supplemented with 10% fetal bovine serum (FBS), 100 U/ml penicillin and 100 μg/ml streptomycin.

Spleens were aseptically removed from the abdominal cavity and minced through a 40 μm nylon cell strainer (Becton Dickinson, Franklin Lakes, New Jersey) with a 5 ml syringe core in 10 ml of PBS. Red blood cells were depleted with Tris-NH_4_Cl lysis buffer. Splenocytes were washed with PBS containing 2 mM EDTA and resuspended in RPMI1640 medium. Splenic CD4^+^ T cells were isolated by magnetic cell sorting using CD4 microbeads (Miltenyi Biotec).

### Antibodies and flow cytometry

Low endotoxin, azide-free anti-mouse CD3 (clone 17A2), CD28 (clone 37.51), PE conjugated anti-mouse CD11b (clone M1/70), Gr-1 (clone RB6-8C5), TER-119 (clone TER-119), B220 (clone RA3-6B2), CD19 (clone 6D5), CD3 (clone 145-2C11), CD11c (clone N418), F4/80 (clone BM8), CD137 (17B5), PE-Cy7 Ly6G (clone RB6-8C5), APC-conjugated CD3 (clone 145-2C11), Ly6C (clone HK1.4), Sca-1 (clone D7), FITC-conjugated CD117 (clone 2B8), eFluor450-conjugated CD4 (clone GK1.5), IL-7R (clone A7R34), eFluor710-conjugated CD8 (clone 53-6.7)and their isotype controls rat IgG2a (clone RTK2758), rat IgG2b (clone RTK4530), Armenian hamster IgG (clone HTK888) were obtained from Biolegend (San Diego, CA, USA).

2 − 3 × 10^5^ cells were stained with specific flourochrome-conjugated antibodies in PBS containing 0.5% FBS and 0.1% sodium azide (FACS buffer) together with mouse FcR blocker (Miltenyi Biotech) for 1 h at 4°C in the dark. Cells were then washed twice and resuspended in 500 μl of FACS buffer. If fixation was required, the cells were fixed with 1% PFA for 1 h at 4°C. Flow cytometry was performed on a Cyan flow cytometer (Dako, Denmark) with Summit software v4.3, or on a BD LSR Fortessa cell analyzer (BDBioscience) and analyzed with Flowjo. Nonspecific staining was controlled by isotype-matched antibodies. Countbright Absolute Counting Beads (Invitrogen) were added to samples for flow cytometry when calculation of absolute cell numbers was performed.

### Colony forming assay

Bone marrow cells from 3 and 12 months old mice were harvested as described above. Cells were resuspended in IMDM at a concentration of 10^6^/ml. 300 μl of cell solution were added to 3 ml of Methocult 3434 (Stem Cell Technologies) for myeloid progenitor detection. 1.1 ml of medium was dispensed to treated culture dish and incubated at 37°C for 7 – 10 days. Types of colonies were determined based on manufacturer's instruction. Duplicates of plates were prepared for each mouse, and at least 3 mice for each strain were analyzed. The morphology of colonies was documented by using a Zeiss Axiovert 40 inverted microscope (Zeiss, Göttingen, Germany) and Canon PowerShot G6 digital camera.

### Isolation of lin^−^ progenitor cells and coculture with activated CD4^+^ T cells

Lin^−^ progenitor cells were isolated by magnetic cell sorting using the mouse lineage cell depletion kit (Miltenyi Biotec). Briefly, fresh bone marrow cells were labeled with a cocktail of biotin-conjugated antibodies against CD5, CD45R, CD11b, Gr-1, Ter-119, 7-4, followed by anti-biotin microbeads. The cell suspension was passed through a LS column in a strong magnetic field and the lineage negative progenitors were collected in the effluent.

Lin^−^ progenitors were resuspended at 10^6^ cells/ml in PBS with 0.1% BSA. Cells were stained with 5 μM CFSE, and 10^5^ CFSE-labeled lin^−^ progenitors were then cocultured with CD4^+^ T cells preactivated by CD3 and CD28 antibodies at the ratio of 1:2. On day 6, the cells were harvested and stained for CD4, CD11b, Ly6G, Ly6C and analyzed by flow cytometry.

### CFSE labeling

Cells were stained with 5 μM CFSE at 37°C for 10 minutes. Incorporation of CFSE dye was stopped by adding 5 volumes of ice-cold medium and incubation on ice for 5 minutes. Cells were then washed with medium and centrifuged at 300g for 10 minutes for three times. CFSE dye was detected by flow cytometry on Cyan flow cytometer (Dako, Denmark) or BD LSRFortessa cell analyzer (BD Bioscience).

### Statistics

Quantitative data are presented as mean ± SD. Statistical significance was determined by a two-tailed unpaired Student's *t*-test. P values less than 0.05 were considered statistically significant.

## SUPPLEMENTARY FIGURE


