# Supplementary material for: CD137 ligand reverse signaling skews hematopoiesis towards myelopoiesis during aging
Source: Aging (Albany NY). 2013 Aug 8;5(9):643–52. doi: 10.18632/aging.100588 (PMC3808697; doi:10.18632/aging.100588)
Supplement: Supplementary file 1 [file aging-05-643-s001.pdf]

30. Sollner L, Shaqireen DOK, Wu JT and Schwarz H. Signal transduction mechanisms of CD137 ligand in human monocytes. Cell Signal. 2007; 19:1899-1908.
31. Kwon BS, Hurtado JC, Lee ZH, Kwack KB, Seo SK, Choi BK, Koller BH, Wolisi G, Broxmeyer HE and Vinay DS. Immune responses in 4-1BB (CD137)-deficient mice. JImmunol. 2002; 168:5483-5490.
32. Pollok KE, Kim YJ, Hurtado J, Zhou Z, Kim KK and Kwon BS. 4-1BB T-cell antigen binds to mature B cells and macrophages, and costimulates anti-mu-primed splenic B cells. EurJImmunol. 1994; 24:367-374.
33. Pauly S, Broll K, Wittmann M, Giegerich G and Schwarz H. CD137 is expressed by follicular dendritic cells and costimulates B lymphocyte activation in germinal centers. JLeukocBiol. 2002; 72:35-42.
34. Middendorp S, Xiao Y, Song JY, Peperzak V, Krijger PH, Jacobs H and Borst J. Mice deficient for CD137 ligand are predisposed to develop germinal center-derived B-cell lymphoma. Blood. 2009; 114:2280-2289.

SUPPLEMENTARY FIGURE

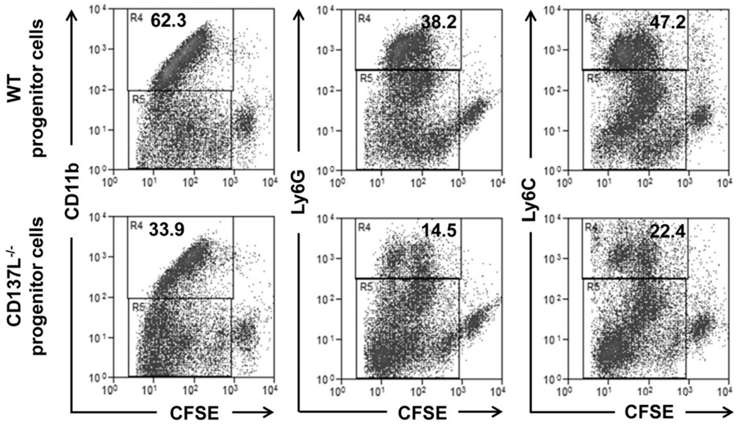

Supplementary Figure 1.
